# What Is the Role of Dietary Inflammation in Severe Mental Illness? A Review of Observational and Experimental Findings

**DOI:** 10.3389/fpsyt.2019.00350

**Published:** 2019-05-15

**Authors:** Joseph Firth, Nicola Veronese, Jack Cotter, Nitin Shivappa, James R. Hebert, Carolyn Ee, Lee Smith, Brendon Stubbs, Sarah E. Jackson, Jerome Sarris

**Affiliations:** ^1^NICM Health Research Institute, Western Sydney University, Westmead, NSW, Australia; ^2^Division of Psychology and Mental Health, Faculty of Biology, Medicine and Health, University of Manchester, Manchester, United Kingdom; ^3^Laboratory of Nutritional Biochemistry, Research Hospital, IRCCS “S. de Bellis”, Castellana Grotte, Italy; ^4^Aging Branch, Neuroscience Institute, National Research Council, Padua, Italy; ^5^Cambridge Cognition, Cambridge, United Kingdom; ^6^Cancer Prevention and Control Program, Arnold School of Public Health, University of South Carolina, Columbia, SC, United States; ^7^Department of Epidemiology and Biostatistics, Arnold School of Public Health, University of South Carolina, Columbia, SC, United States; ^8^Connecting Health Innovations LLC, Columbia, SC, United States; ^9^Cambridge Centre for Sport and Exercise Sciences, Anglia Ruskin University, Cambridge, United Kingdom; ^10^Physiotherapy Department, South London and Maudsley NHS Foundation Trust, London, United Kingdom; ^11^Health Service and Population Research Department, Institute of Psychiatry, Psychology and Neuroscience, King’s College London, London, United Kingdom; ^12^Department of Behavioural Science and Health, University College London, London, United Kingdom; ^13^Department of Psychiatry, University of Melbourne, The Melbourne Clinic Professorial Unit, Melbourne, VIC, Australia

**Keywords:** nutrition, schizophrenia, bipolar disorder, nutrients, vitamin

## Abstract

Severe mental illnesses (SMI), including major depressive disorder, bipolar disorder, and schizophrenia, are associated with increased inflammation. Given diet’s role in modulating inflammatory processes, excessive calorie-dense, nutrient-deficient processed food intake may contribute toward the heightened inflammation observed in SMI. This review assesses the evidence from observational and experimental studies to investigate how diet may affect physical and mental health outcomes in SMI through inflammation-related pathways. Cross-sectional studies indicate that individuals with SMI, particularly schizophrenia, consume more pro-inflammatory foods and fewer anti-inflammatory nutrients than the general population. Cohort studies indicate that high levels of dietary inflammation are associated with increased risk of developing depression, but there is currently a lack of evidence for schizophrenia or bipolar disorder. Randomized controlled trials show that dietary interventions improve symptoms of depression, but none have tested the extent to which these benefits are due to changes in inflammation. This review summarizes evidence on dietary inflammation in SMI, explores the directionality of these links, and discusses the potential use of targeted nutritional interventions for improving psychological well-being and physical health outcomes in SMI. Establishing the extent to which diet explains elevated levels of inflammatory markers observed in SMI is a priority for future research.

## Introduction and Aims

Recent meta-analyses have confirmed that severe mental illnesses (SMI), including major depressive disorder (MDD), bipolar disorder, and schizophrenia, are associated with increased levels of both peripheral inflammatory markers (1) and systemic inflammation (2). Additionally, heightened inflammation could present a novel treatment target for MDD, given that the anti-depressant efficacy of various pharmacological and lifestyle interventions appears to be associated with reductions in inflammation (3, 4). In schizophrenia, the evidence for antipsychotics altering inflammatory markers is mixed (1, 5), although there is some preliminary evidence to indicate that various adjunctive interventions may confer beneficial effects through reducing inflammatory status (6, 7).

Calorie-dense diets that are high in saturated fats and simple carbohydrates appear to increase peripheral inflammatory markers, whereas diets high in fiber and vegetables reduce inflammation (8–12). Systematic reviews of dietary patterns in people with SMI have shown elevated intakes of sugar-sweetened soft drinks, refined grains, and processed meat are common in this population (13, 14). However, the degree to which these dietary patterns heighten inflammation in SMI, and the potential impact on physical and mental health outcomes, is relatively unexplored. This comprehensive review brings together the evidence from cross-sectional, longitudinal, and experimental studies on this topic to:

- Examine the extent to which inflammatory potential of the diet (hereafter referred to as “dietary inflammation”) is elevated in SMI populations;
- Explore the directionality of the links between dietary inflammation and symptoms of SMI;
- Discuss the existing evidence for the use of nutritional interventions for improving health outcomes in SMI and how these effects may act through inflammatory pathways.

## Food Intake and Dietary Inflammation in People with Severe Mental Illnesses

**Figure 1:**
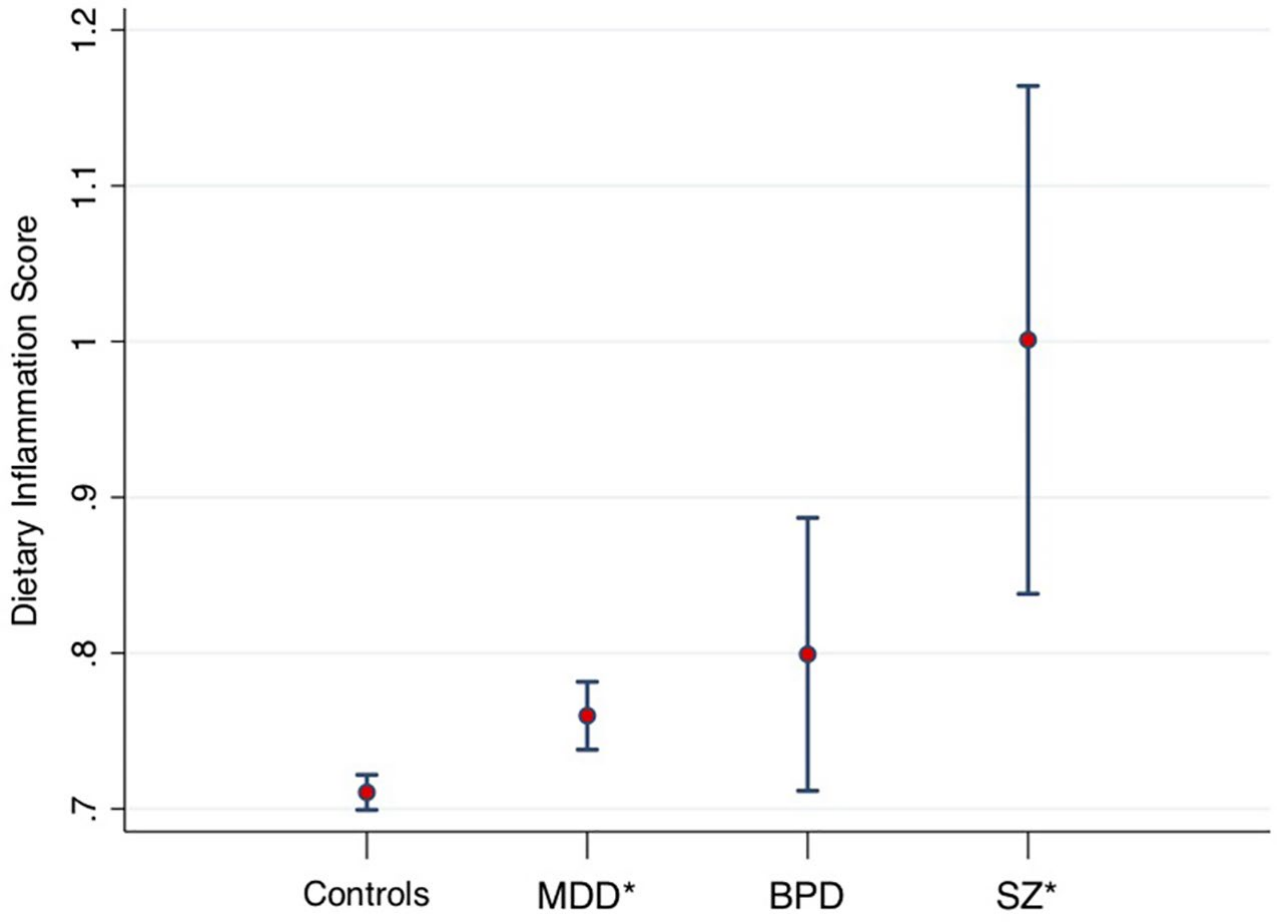
Dietary Inflammatory Index (DII) scores from 53,270 healthy controls, compared to major depressive disorder (MDD) (n = 14,422), bipolar disorders (BPD, n = 933), and schizophrenia (SZ, n = 254). Midpoint shows adjusted means. Error bars show 95% confidence intervals. *Statistically significant difference compared to healthy controls. Data derived from Firth et al. (15).

A recent large-scale study of the UK Biobank (15) compared the macro- and micro-nutrient intake of individuals with diagnosed MDD (n = 14,619), bipolar disorder (n = 952), and schizophrenia (n = 262) to healthy controls (n = 54,010), showing that people with SMI consumed significantly more carbohydrate, sugar, fat, and saturated fat than healthy controls (all p < 0.001), even when controlling for age, gender, education, BMI, social deprivation, and ethnicity. The study also examined the inflammatory potential of food intakes of individuals with SMI compared with the general population using the “Dietary Inflammatory Index” (DII^®^). The DII is a literature-derived, population-based measure, which provides an estimate of the inflammatory potential of an individual’s diet from up to 45 individual food parameters (16). DII scores have been validated against various blood markers of inflammatory status across a number of different populations (17–21). The DII scores in SMI samples in the UK Biobank are displayed in **Figure 1** [derived from Firth et al. (15)], adjusted for age, gender, and total energy intake. These data show highly elevated dietary inflammation in individuals with schizophrenia, along with smaller, but significantly increased, levels of dietary inflammation in individuals with MDD (all p < 0.01). Although dietary inflammation in the bipolar disorder group was similarly larger than healthy controls (p = 0.03), this difference was reduced to a marginally non-significant trend after adjusting for BMI and socioeconomic status (p = 0.07).

It is interesting to note that despite the vast number of studies examining elevated levels of peripheral inflammation observed across all classes of SMI (1), none have accurately controlled for the potential confounding factor of diet. Furthermore, a priority for future research is to validate the accuracy of dietary reporting in SMI. Interestingly, previous research comparing other lifestyle factors (i.e., physical activity) using objective against self-report measures in SMI have shown that people with schizophrenia significantly overestimate health behaviors compared with the general population (22). Therefore, replication of these findings, using validated measures in SMI alongside blood markers of inflammation, is required to establish how diet may relate to inflammation in SMI.

Along with poor mental health, people with SMI experience drastic inequalities in physical health, including elevated rates of obesity, diabetes, and cardiometabolic disorders, ultimately contributing to a reduced life expectancy of around 20 years (23). Given the clear causal links between dietary inflammation and these health outcomes established in the general population (10–12), and the established benefits of dietary interventions for physical health in SMI (24), it is reasonable to explore dietary inflammation as one risk factor driving some of the physical health inequalities observed in this population. Indeed, the highest levels of dietary inflammation are observed in schizophrenia: a group that also experiences significantly worse physical health outcomes than other classes of SMI (25, 26). Poor dietary quality associated with schizophrenia may even be driven by side effects of antipsychotic medications, which may increase appetite through interfering with the “hunger hormone,” ghrelin (27). Clearly, there is an urgent need for future research to determine the mechanisms through which poor diet may be driving adverse health outcomes in people with SMI. This line of investigation will provide novel insights into the etiology of the physical health inequalities observed in this population and has the potential to inform clinical care.

A key limitation of the current literature is a lack of large-scale data on dietary patterns among young people with SMI, thus making it difficult to determine whether poor diet precedes the onset of mental illness, or vice versa. In the general population, data suggest that younger people tend to have worse diets than older adults (28). This also may apply to SMI populations, as nutritional deficits in psychosis are evident even prior to antipsychotic treatment (29). Thus, in the following section, we review the prospective studies examining links between high levels of dietary inflammation and the subsequent onset of mental illness.

## Prospective Associations Between Dietary Inflammation and Psychiatric Symptoms

Poor nutrition has been implicated in the onset and persistence of psychiatric disorders (30). In general, cohort studies have shown that dietary patterns, such as a Mediterranean diet, which is rich in fruits, vegetables, olive oil, and legumes, may be protective against mental health disorders (31–38). By contrast, increased risk of mental disorders has been observed with dietary patterns, such as the Western diet, characterized by high intake of saturated fat and refined carbohydrates (33, 39–41).

Inflammation presents one feasible mechanism through which diet may affect the risk of mental disorders. This is supported by multiple cohort studies showing that higher DII scores are associated with increased risk of depression (42–49). Combining all longitudinal data on this topic (including 77,420 participants from seven different studies), a recent meta-analysis confirmed that higher levels of dietary inflammation were associated with 31% increased risk of depression over the 5- to 13-year follow-up period (50). This meta-analysis also found that pro-inflammatory diets were more strongly associated with depression among females than males (50), although significant relationships were observed for both sexes.

Despite these positive findings on links between depression and dietary inflammation calculated from self-report measures, future research must establish if these relationships are mediated by biological markers of inflammatory status. Although a number of studies have found joint relationships between dietary inflammation, inflammatory markers, and depressive symptoms (51–53), those findings are inconsistent with other results showing that dietary patterns associated with heightened inflammatory markers do not consistently predict depression scores (54).

Currently, there is an urgent need for longitudinal studies to assess how dietary inflammation is related to the onset of other classes of SMI, because there is currently no strong evidence linking dietary inflammation with risk of bipolar disorder or schizophrenia. As the effects of dietary inflammation on mental health are also observed in adolescence (51), when the majority of SMIs first arise (55), the potential impact that this may have on risk of bipolar and psychotic disorders is worthy of further examination.

Along with clinical symptoms, people with SMI (and particularly schizophrenia) also display a range of cognitive deficits (56–58), which impede daily functioning (59, 60), and are not treated by psychotropic medications (61, 62). There is an emerging literature suggesting that elevated peripheral inflammatory markers are associated with deficits in cognitive function among patients with psychiatric disorders (1, 63). Though the specific mechanisms underlying this association remain unclear, chronic and acute inflammation is thought to have a number of detrimental effects on brain structure and function, which, in turn, appear to adversely affect cognitive performance (64–66).

Poor diet and obesity also have a well-established link with cognitive dysfunction (67, 68). There is mounting evidence that these associations may be mediated by inflammatory processes (69), suggesting that diet has the potential to act as a modifiable risk factor for cognitive dysfunction both in clinical and non-clinical populations. Much of the work investigating the association between diet, inflammation, and cognition has come from a series of cross-sectional and longitudinal studies in older adults, which indicate that diets with high inflammatory potential may be associated with accelerated cognitive decline and reduced brain volume (70–72). Considering the high levels of dietary inflammation and cognitive deficits observed in SMI, along with indicated relationships between cognitive functioning and diet in other populations, this area presents a promising avenue for future research (73).

## Experimental Manipulation of Dietary Inflammation: Can we Make a Difference to Mental Health?

A recent meta-analysis examined the effects of dietary interventions on mental health in 16 randomized controlled trials (RCTs) of 45,826 participants (74). Dietary improvement significantly reduced symptoms of depression [*g* = 0.275; 95% confidence interval (CI), 0.10–0.45; *p* = 0.002], with no changes in anxiety observed. Interestingly, similar degrees of benefit for depressive symptoms were observed from all the different dietary approaches trialed; dietary interventions primarily designed to improve nutrition [e.g., the Mediterranean diet, which is typically linked with anti-inflammatory effects (11)] were no more beneficial for mental health than those aimed at reducing bodyweight or decreasing dietary fat intake (74). This may be because, even without increasing anti-inflammatory nutrient intake, weight-loss and changes in energy balance can reduce inflammation through reducing excess adipose tissue, which is associated with heightened inflammation (75, 76).

However, 15 of the 16 RCTs in this meta-analysis only examined effects on depressive symptoms in samples with “sub-clinical” depression (i.e., samples without a confirmed diagnosis of MDD). However, the single trial conducted in clinically depressed participants (75) observed large reductions in depressive symptoms from a 12-week modified Mediterranean diet, with 32.3% of participants achieving remission from the dietary intervention versus 8.0% in the social support control condition (*p* = 0.028). Subsequent RCTs have replicated these findings of the Mediterranean diet reducing symptoms in people with moderate to severe depression (76). As a meta-analysis of 50 studies (10) has shown, the Mediterranean diet significantly reduces inflammatory markers in other (i.e., non-psychiatric) populations, and it is possible that the benefits in people with depression are linked to the anti-inflammatory effects. However, this has yet to be assessed, as no studies have measured changes in inflammation following dietary interventions in depression. Furthermore, there is currently no experimental evidence showing beneficial effects of dietary interventions on inflammation and mental health in schizophrenia or bipolar disorder.

Nonetheless, RCTs of individual nutrient-based supplements (nutraceuticals) have provided valuable insights into how nutrition can influence mental health in SMI through inflammatory pathways. For instance, in an RCT of 155 individuals with MDD, Rapaport et al. (77) found that patients with baseline elevated markers of inflammation were significantly more responsive to omega-3 treatment (mediated, in principle, *via* eicosapentenoic acid) (78). The antidepressant effects of omega-3 fatty acids working through the reduction of inflammation also were implicated in a seminal study by Su (79). This study examined depression in people with hepatitis C, undergoing interferon (IFN) alpha therapy, which commonly induces depressive symptoms due to its inflammatory effects (80). However, Su et al. (79) found that omega-3 supplementation reduced the risk of developing depression following INF-a treatment. Other nutrients, such as folate, have also been found to reduce depression in people with high levels of inflammation (81), again indicating these adjunctive treatments may confer symptomatic benefits through inflammatory pathways.

Beyond MDD, there are preliminary data from RCTs suggesting that anti-inflammatory nutrients, such as omega-3 and folate-based compounds, may also be effective for other SMIs, including bipolar disorder and schizophrenia (82). Because inflammation is particularly elevated during onset of psychotic disorders, these adjunctive treatments may have neuroprotective effects in the early stages of illness among young people (83, 84), potentially improving cognitive outcomes for some patients. However, the extent to which their effects are due specifically to their anti-inflammatory properties is not fully ascertained.

## Conclusions and Future Research

**Figure 2:**
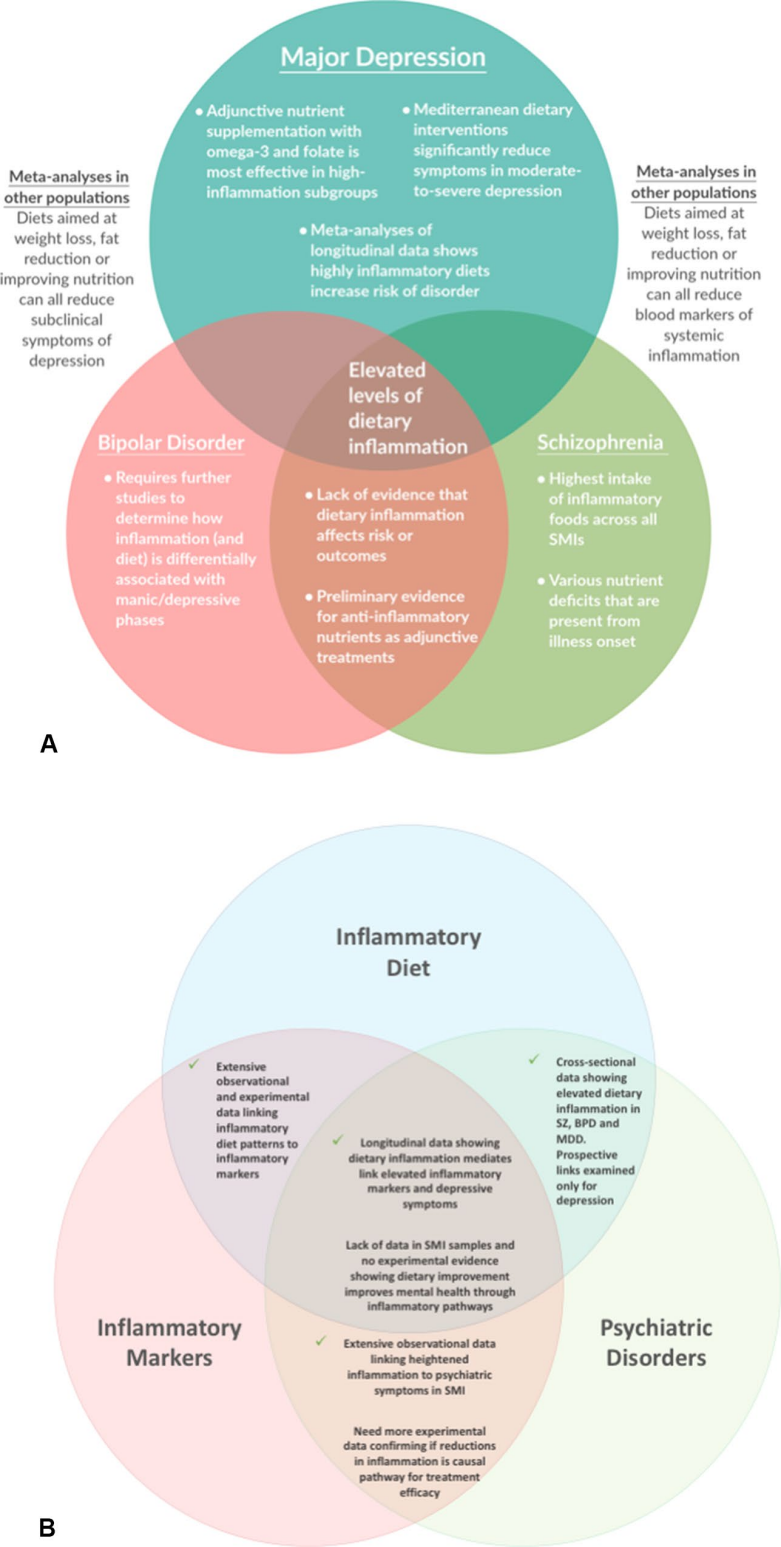
Key Findings and Future Questions: A map of the evidence for the role of dietary inflammation in severe mental illnesses (SMI), with regard to **(A)** different conditions, and **(B)** different aspects of the interaction between dietary inflammation, inflammatory markers, and psychiatric disorders.

The current evidence from human studies examining the role of dietary inflammation in SMI are shown in **Figure 2**. The cross-sectional literature provides consistent evidence that individuals with SMI consume more pro-inflammatory foods than the general population, and fewer anti-inflammatory nutrients—which may contribute toward the heightened levels of inflammatory markers observed in SMI. In the few studies that have compared different classes of SMI, the highest dietary risks are observed among people with schizophrenia (who also have the most severe disparities in physical health, compared with other mental disorders). However, the bulk of both the observational and experimental studies examining the links between dietary inflammation and mental health have been conducted in depression (see **Figure 2A**)—with a relative dearth of evidence in other disorders. Therefore, there is now a need for researchers and clinicians to build upon the existing evidence in MDD and give further attention to the impact of dietary inflammation in schizophrenia and bipolar disorder and explore the potential benefits of dietary modification for these populations.

The longitudinal studies now provide population-scale data showing that high levels of dietary inflammation are associated with increased the likelihood of developing depression over time. However, there is little evidence to suggest that this also applies to schizophrenia or bipolar disorder. Alongside this, a key remaining question in this field (which can only be addressed by experimental studies), is: “Can reducing dietary inflammation make a difference?” or, more specifically, “Is it possible that dietary modification can reduce inflammation and thus improve symptoms in people with SMI?”

Currently, there is no experimental evidence to show that a specific “anti-inflammatory” diet influences psychiatric symptoms of schizophrenia or bipolar disorder. Furthermore, whereas RCTs and meta-analyses have recently shown that dietary improvement reduces symptoms of depression (in both clinical and non-clinical populations), the extent to which this is due to anti-inflammatory effects of dietary interventions has not been assessed. Nonetheless, some evidence from nutraceutical trials suggests that certain anti-inflammatory nutrients may provide adjunctive treatments for subgroups of individuals with mental health conditions with particularly elevated levels of inflammation.

With regard to whole-diet interventions, it is interesting to consider the prevalent finding that the weight loss, fat reduction, or Mediterranean diets trialed so far all appear to confer similar beneficial effects on depressive symptoms. Whereas this may indicate a lack of specificity, it should be acknowledged that each of these interventions, although differing in stated aims, generally have some key factors in common. Specifically, all of these interventions generally involve decreasing the amount of refined, processed calorie-dense foods, while increasing intake of nutrient-dense natural-occurring fiber and vegetables. Therefore, the general equivalence across difference types of diets could ultimately produce an encouraging message, suggesting that highly specific or specialized diets are perhaps unnecessary for the average individuals, as adhering to very simple and universally accepted dietary advice appears to be equally beneficial for psychological well-being—and sufficient for avoiding the potentially deleterious effects on mental health of a “junk food” diet. To provide greater insight on this, future research should attempt to elucidate the specific mechanisms through which the dietary impacts upon inflammation to influence mental health. For instance, hyperglycemia and hyperinsulinemia after a meal of refined starches and sugars may promote inflammation by increasing production of free radicals and pro inflammatory cytokines (85, 86), whereas high levels of saturated fat intake decrease production of short chain fatty acids such as butyrate, which have anti-inflammatory properties (87). Alongside these nutritional factors, obesity and excess adipose tissue themselves directly heighten inflammation—suggesting that attenuating these adverse states of health through calorie restriction and low-fat diets could reduce inflammatory status and thus improve psychological well-being (74, 88). Along with reducing dietary patterns with inflammatory potential, the adequate intake of beneficial nutrients is another mechanism through which “healthy diets” improve inflammatory profiles and support mental health. For example, various vitamins and minerals have been shown to modulate the “kyrenuine pathway” (89), which regulates the immune system, particularly with regard neurotrophic factor production, NMDA receptor signaling, and glutamatergic neurotransmission—all of which are implicated in inflammatory hypotheses of SMI (90).

A further emerging pathway through which inflammatory potential of the diet may induce depressive symptoms is by interacting with the gut–brain axis and affecting the gut microbiome (91). However, the role of individual nutrients on modifying the microbiome is still poorly understood, as are the exact mechanisms by which the gut microbiome itself affects mental health (92).

Further investment in human trials is now required to establish the feasibility and efficacy of dietary improvement as an intervention for improving physical and mental health across different classes of SMI. Additionally, future trials should aim to measure peripheral and central inflammation before and after dietary interventions in SMI. In this way, researchers could apply subgroup and mediation analyses to examine how the potential benefits of nutrition interventions are related to changes in inflammatory status. Ultimately, this line of investigation could shed new light on the interface between physical and mental health in people with SMI, along with presenting novel interventions and adjunctive treatments for improving psychological well-being and tackling the poor cardiometabolic health observed in this underserved population.

## Author Contributions

All authors contributed to the conception, development, and writing of this mini-review. All authors have approved the final paper.

## Conflict of Interest Statement

JC is an employee of Cambridge Cognition Ltd. JH owns controlling interest in Connecting Health Innovations LLC (CHI), a company planning to license the right to his invention of the Dietary Inflammatory Index (DII^®^) from the University of South Carolina in order to develop computer and smart phone applications for patient counseling and dietary intervention in clinical settings. NS is an employee of CHI. The subject matter of this paper will not have any direct bearing on that work, nor has that activity exerted any influence on this paper. JF is supported by a Blackmores Institute Fellowship. JF, JS, and CE declare that as a medical research institute, NICM Health Research Institute receives research grants and donations from foundations, universities, government agencies, and industry. Sponsors and donors provide untied and tied funding for work to advance the vision and mission of the Institute. CE declares that she is an integrative GP. The remaining authors declare that the research was conducted in the absence of any commercial or financial relationships that could be construed as a potential conflict of interest.
